# Comparison of Physicochemical Characteristics and Anticoagulant Activities of Polysaccharides from Three Sea Cucumbers

**DOI:** 10.3390/md11020399

**Published:** 2013-02-05

**Authors:** Lan Luo, Mingyi Wu, Li Xu, Wu Lian, Jingying Xiang, Feng Lu, Na Gao, Chuang Xiao, Shengmin Wang, Jinhua Zhao

**Affiliations:** 1 State Key Laboratory of Phytochemistry and Plant Resources in West China, Kunming Institute of Botany, Chinese Academy of Sciences, Kunming 650201, China; E-Mails: luolan_41@163.com (L.L.); mingyiwu_tju@yahoo.com (M.W.); xulib@mail.kib.ac.cn (L.X.); lw3662410@yahoo.com.cn (W.L.); wind137@126.com (J.X.); lu.fengai@163.com (F.L.); gn2008.happy@163.com (N.G.); xchuang@mail2.sysu.edu.cn (C.X.); 2 School of Life Science and Engineering, Southwest Jiaotong University, Chengdu 611756, China; E-Mail: wangshengmin@sohu.com

**Keywords:** sea cucumber, polysaccharide, fucosylated chondroitin sulfate, sulfated fucan, glucan, anticoagulant activity

## Abstract

In order to search for sulfated polysaccharides in different invertebrate connective tissues and to examine their biological activities, we have isolated three types of polysaccharides from the body wall of the three sea cucumbers *Holothuria edulis*, *Apostichopus japonicas* and *Holothuria nobilis*. The physicochemical properties and anticoagulant activities of these polysaccharides were examined and compared. The chemical composition analysis and nuclear magnetic resonance (NMR) analysis indicate that two types of polysaccharides, sulfated fucan and fucosylated chondroitin sulfate (FuCS), were found in all of the three species and in addition a neutral glycan was observed in *H. edulis*. The neutral α-glucan was firstly obtained from sea cucumber. The same type of polysaccharides from different species of sea cucumbers have similar physicochemical properties and anticoagulant activities, but those of different types of glycans are significantly different, possibly due to their different monosaccharide compositions, electric charges and average molecular weights. The FuCSs have stronger anticoagulant activities than the sulfated fucans, although the molecular sizes of the FuCSs are lower than those of the sulfated fucans, whereas the neutral glucan has no activity, as expected from the absence of sulfate. Thus, anticoagulant activities of the different type of polysaccharides are likely to relate to monosaccharide composition and sulfate content. Preliminary analysis suggests that the sulfation patterns of the FuCSs may result in the difference in anticoagulant activities. Our data could help elucidate the structure-activity relationship of the sea cucumber polysaccharides.

## 1. Introduction

Thromboembolic diseases continue to be the leading cause of death throughout the world [1]. Due to aging and the high incidence of thrombosis, unfractionated heparin (UFH) and low-molecular-weight heparins (LMWHs) have been the cornerstone in antithrombotic treatment and prophylaxis for the last 60 years. However, the heparin source is limited. It is obtained from porcine intestine or bovine lung, and contamination of samples with pathogens is a serious concern [2]. Therefore, there is a great need for new compounds from natural sources, and marine organisms are an abundant source of sulfated polysaccharides with anticoagulant and antithrombotic activities [3,4,5,6].

Sea cucumbers are marine invertebrates that have gained popularity among researchers in recent decades, not only due to their nutritive value, but also due to their potential health benefits and therapeutic uses [4]. An extensive literature survey reveals that sea cucumber has a long history as a traditional food and folk medicine [4]. For example, bioactives isolated from sea cucumber, novel sulfated polysaccharides such as fucosylated chondroitin sulfate (FuCS) and sulfated fucan, have shown anticoagulant [3,6,7] and antithrombotic activities [8]. They have clear advantages over previously known anticoagulants such as heparin, since they are not obtained from mammals, occur at high concentrations in an abundant invertebrate and retain the serpin-independent anticoagulant activity [3]. Thus, there is increasing interest in the anticoagulant activities of these sulfated polysaccharides. 

Previous studies have suggested that anticoagulant activity mostly depends on the molecular size of the polysaccharides and also relates to monosaccharide composition, sulfate content and position [9,10,11,12]. The anticoagulant activity of the FuCS from the sea cucumber *Thelenota ananas* as measured by the activated partial thromboplastin time assay varies in proportion to the molecular weight following a logarithmic-like function [10]. Heparin cofactor II-mediated antithrombin activity of a sulfated fucan is dependent on both its sulfate content and molecular weight [11]. The inhibitory effects of fucans on coagulation are dependent on their sulfation degree and molecular weight [12]. 

Very recently, we have isolated a fucosylated chondroitin sulfate from the body wall of the sea cucumber *T. ananas* and elucidated its chemical structure and anticoagulant activity [10,13,14]. In order to examine the distribution of the sea cucumber polysaccharides and to compare their physicochemical characteristics and biological activities, we have now expanded our studies to include more species of sea cucumber, namely *Holothuria edulis*, *Apostichopus japonicas* and *Holothuria nobilis*. The polysaccharides obtained from these sea cucumbers are three FuCSs, three sulfated fucans and a neutral glucan, respectively. We further found that the polysaccharides from different sea cucumber species have distinctive physicochemical characteristics. The results where the physicochemical characteristics have been fully characterized by a systematic analysis of nuclear magnetic resonance (NMR), chemical composition, electric charge, average molecular weight and intrinsic viscosity, are valuable tools to trace the relationship between structures *versus* anticoagulant activities of the sea cucumber polysaccharides.

## 2. Results and Discussion

### 2.1. Extraction and Purification

**Figure 1:**
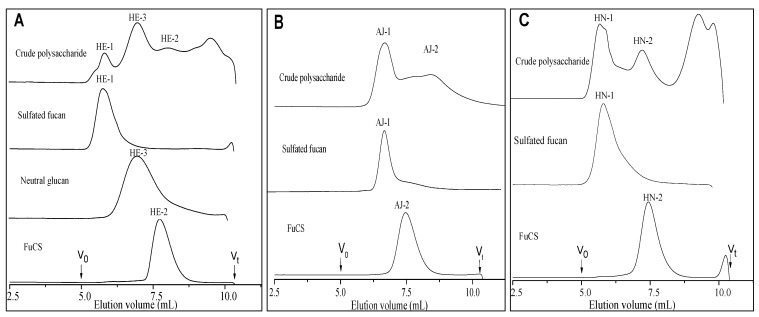
High-performance gel permeation chromatography (HPGPC) profiles of crude polysaccharides, sulfated fucans, fucosylated chondroitin sulfates and neutral glucan from the three species of sea cucumbers *H. edulis* (*Panel **A***), *A. japonicas* (*Panel **B***) and *H*. *nobilis* (*Panel **C***).

The yields of crude polysaccharides isolated from the three species of sea cucumbers, *H*. *edulis* (HE), *A. japonicas* (AJ) and *H*. *nobilis* (HN), were 6.3%, 2.6% and 6.3% by dry weight, respectively. The high-performance gel permeation chromatography (HPGPC) of the extracted polysaccharides on a Shodex OH-pak SB-804 HQ column showed three peaks for *H*. *edulis*, whereas two peaks for each of the sea cucumbers *A. japonicas* and *H*. *nobilis* (Figure 1) were found, indicating a difference in molecular weight.

**Figure 2:**
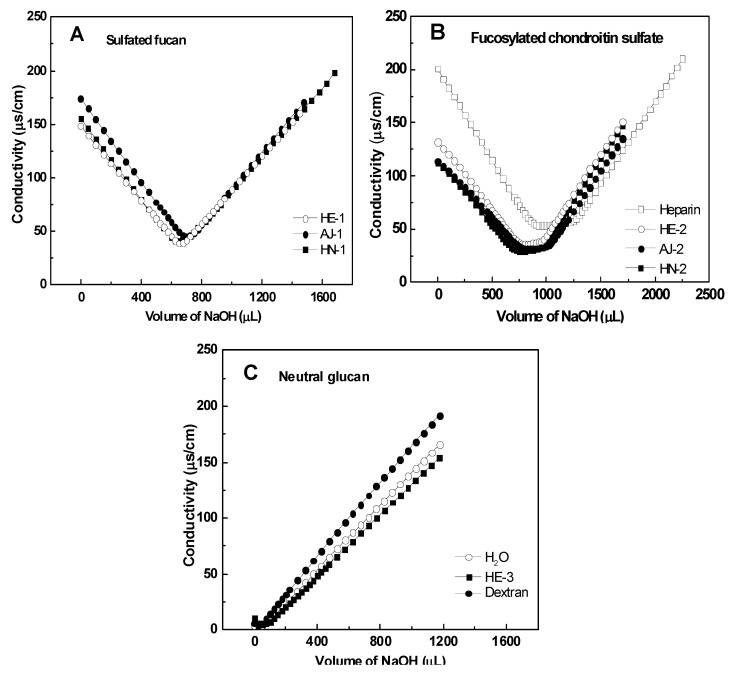
Conductimetric titration curves of samples of sulfated fucans (*Panel **A***), fucosylated chondroitin sulfates (*Panel **B***) and neutral glucan (*Panel **C***) from three sea cucumbers.

Sulfated polysaccharides were purified from the body wall of three species of sea cucumbers. Purification was achieved by Sephadex G-100 and anion exchange chromatography on a DEAE-Sepharose FF column. The crude polysaccharides were separated into three or two major fractions, which were eluted with different NaCl concentrations. Based on their chemical compositions, NMR analysis data and conductimetric titration curves (Table 1, Figure 2 and see below for discussion), we found that the three species of sea cucumbers contained sulfated fucans (peaks HE-1, AJ-1 and HN-1 in the Figure 1) and fucosylated chondroitin sulfates (peaks HE-2, AJ-2 and HN-2 in the Figure 1) as has also been reported for other sea cucumber species [3,13,15,16,17,18,19,20,21], but surprisingly, the sea cucumber *H*. *edulis* contains a neutral glucan (peak HE-3 in the Figure 1, Table 1). In addition, analysis of sulfated fucans and fucosylated chondroitin sulfates by anion exchange chromatography on a DEAE Sepharose FF column confirms the high negative charge densities of the two polysaccharides (not shown), as expected from analysis of the sulfate and carboxyl by the conductometric titration (Figure 2). 

The purities of these polysaccharides were confirmed by gel filtration chromatography on a Shodex OH-pak SB-804 HQ column. The results show they each migrate as a single homogeneous peak (Figure 1) and do not have any ultraviolet absorption near 260 or 280 nm by the measurement of an UV-detector, indicating no contaminants such as protein and peptide. The contents of the two sulfated polysaccharides are different: The fucosylated chondroitin sulfates are less abundant than sulfated fucans in the three species of sea cucumbers by HPGPC profile analysis of the crude polysaccharides (Figure 1).

**Table 1:** Chemical composition and physicochemical properties of polysaccharides from the body wall of three sea cucumbers.

**Samples**	**Chemical composition (molar ratios) ^a^**						**Average molecular weight **			**Specific rotation**	**[η]**	***Κ_H_***	***Κ_K_***
	GlcA	GalNAc	Fuc	Glc	OSO_3_^−^/COO^−^	SO_3_^−^/Fuc	*M_n_*	*M_w_*	*M_w_*/*M_n_*		**(mL·g** **^−1^)**	**(×10** **^−4^)**	**(×10** **^−4^)**
**Sulfated fucans**													
HE-1	<0.01	<0.01	1.00	<0.01	/	0.53	517,900	615,500	1.19	−181.4°	605.6	2.12	−1.49
AJ-1	<0.01	<0.01	1.00	<0.01	/	0.57	225,900	419,900	1.86	−181.5°	208.6	1.98	−2.37
HN-1	<0.01	<0.01	1.00	<0.01	/	0.51	289,400	475,800	1.64	−193.4°	428.0	2.56	−1.63
**Fucosylated** ** chondroitin sulfates and a standard polysaccharide**													
HE-2	1	1.28	0.82 ^b^	<0.01	3.50	ND ^c^	41,340	51,090	1.24	−37.9°	24.6	−8.71	−12.62
AJ-2	1	1.05	1.03 ^b^	<0.01	2.96	ND	46,760	56,820	1.22	−56.2°	32.5	−20.95	−11.50
HN-2	1	0.96	0.82 ^b^	<0.01	2.99	ND	42,460	55,320	1.30	−45.9°	34.0	−10.48	−14.06
Heparin	ND	ND	ND	<0.01	2.04	ND	20,230	26,260	1.30	48.2°	24.5	−11.60	−15.59
**Neutral glucan**													
HE-3	<0.01	<0.01	<0.01	1.00	/	/	197,900	253,300	1.28	170.2°	0.8	15397	15212

^a^ GlcA: glucuronic acid; GalNAc: *N*-acetyl-β-D-galactosamin; Fuc: α-L-Fucose; Glc: D-glucose. ^b^ The ratios of GlcA and GalNAc were determined by the chemical methods (Experimental Section), and the ratios of GalNAc and Fuc were determined by their –CH_3_ integrals analysis of ^1^H-NMR spectra. ^c^ ND: Not determined.

### 2.2. Physicochemical Characteristics

#### 2.2.1. Chemical Composition and Analysis of Sulfate and Carboxyl Groups

**Figure S1:**
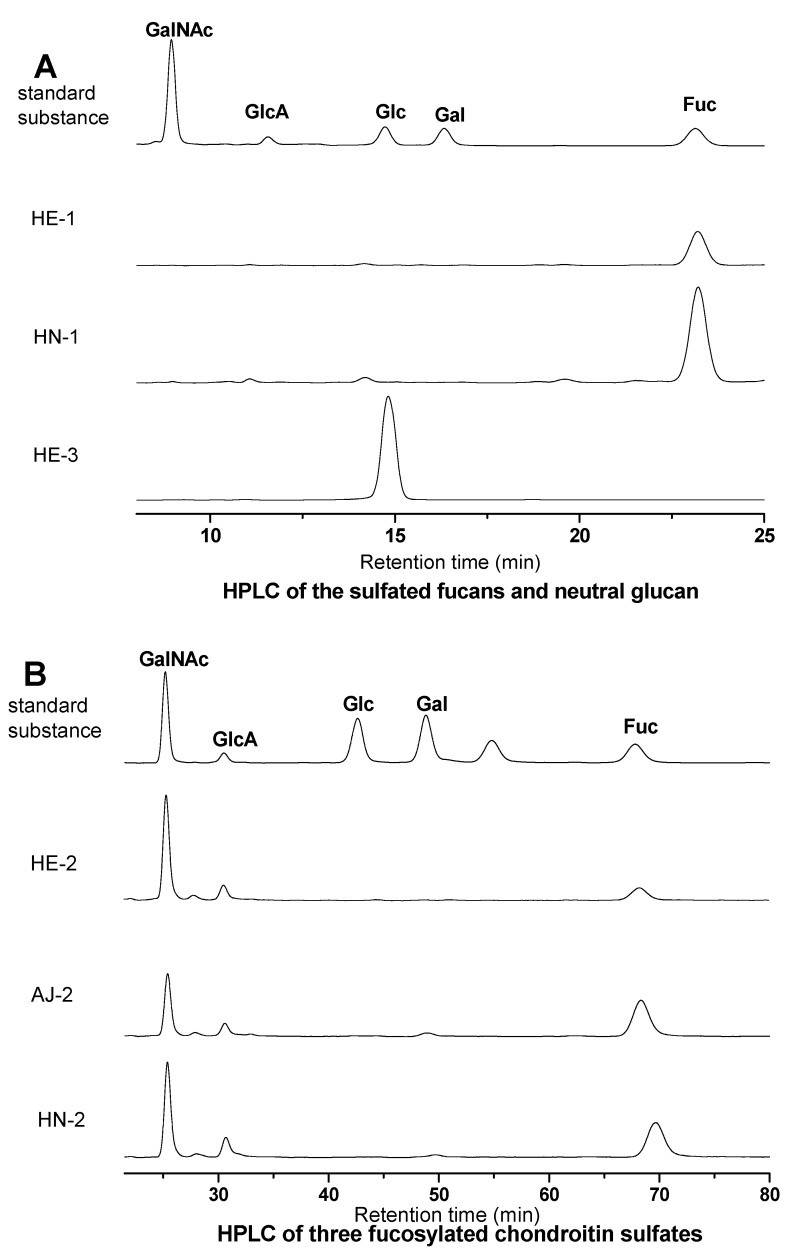
HPLC of the sulfated fucans, neutral glucan (*Panel **A***) and the fucosylated chondroitin sulfates (*Panel **B***).

These polysaccharide fractions have been characterized by different analytical techniques to compare their physicochemical properties. The chemical analysis shows that they have various monosaccharide compositions (Table 1, Figure S1). Fractions HE-1, AJ-1 and HN-1 contain fucose as the only sugar with a high content of sulfate ester, whereas the polysaccharide fractions HE-2, AJ-2 and HN-2 are composed of three monosaccharides, namely glucuronic acid (GlcA), *N*-acetylgalactosamine (GalNAc) and fucose (Figure S1). Fractions HE-2, AJ-2 and HN-2 can be assigned to be the chondroitin sulfate as GlcA and GalNAc are in around 1:0.96–1:1.28 ratios (Table 1), possibly similar to the chondroitin sulfate backbone structure. The fucose and sulfate contents of these FuCSs obtained from different sea cucumbers vary from each other, which may account for their structure complexity. Cleavage of the sea cucumber FuCSs by digestion with specific chondroitin lyases or by deamination with nitrous acid was followed by HPGPC. These polysaccharides with high molecular mass were not reduced by digestionwith chondroitin AC or ABC lyase or by deamination with nitrous acid (not shown), possibly because their sulfated fucose branches may be linked to Position 3 of the GlcA residues [13,17,18,19,20]. The results are in agreement with previous reports on the FuCSs from others species of sea cucumbers [17,18,19].

Chemical analysis of the fraction HE-3 (Table 1) reveals glucose as the only sugar without a content of sulfate ester. Thus, preliminary analysis indicates that the fraction HE-3 may be a neutral glucan.

Among other information needed, the contents of sulfate and uronic groups are essential to evaluate the charge distribution along the polyelectrolyte chain [22]. Thus, the sulfate and carboxyl of the sea cucumber polysaccharide were measured by conductometric titration. The conductance curves obtained by titrating the acid form of the polysaccharides with a strong base (0.02 M NaOH) are shown in Figure 2. Clearly, these curves differ in inflexion point. Extrapolation of the three branches of the conductimetric curve of the FuCS gives two intersections, the first corresponding to the equivalence point of the sulfates (SO_3_^−^), and the second to that of the carboxyls (COO^−^) (Figure 2B), which is similar to standard heparin. The curve of the sulfated fucan has only one inflexion point, corresponding to the sulfates, but not carboxyls (Figure 2A); whereas that of the glucan appears to have no inflection point, similar to a standard dextran, indicating the absence of charge (Figure 2C).

Data for the SO_3_^−^ and COO^−^ determinations are given in Table 1. The three sulfated fucans from different species of sea cucumbers have the same sulfate: sugar molar ratio, close to 0.55. The molar ratios are different from those of the sulfate fucans from *Ludwigothurea grisea* [15] and *Stichopus japonicas* [16]. The three fucosylated chondroitin sulfates have different SO_3_^−^ to COO^−^ molar ratio (3.50, 2.99 and 2.96, respectively), more than the FuCS from *L*. *grisea* [17], but less than the FuCS from *T*. *ananas* [10] and *S*. *japonicas* [18]. These variations may also reflect other important structural differences [13].

#### 2.2.2. Specific Optical Rotation

In certain wavelength and temperature conditions, the optical phenomenon of an optically active substance reflects the specific structure and specific rotation changes along with the structure changes. Table 1 shows the specific optical rotation of the purified sea cucumber polysaccharides. The different types of polysaccharides have different specific rotations, but the same type of polysaccharides from different sea cucumbers have similar specific rotations, as expected from the different monosaccharide composition (Table 1). The strongly negative specific rotation of the sulfated fucans (Table 1) is compatible with residues of α-L-fucopyranose [21]. The specific rotations of the FuCSs are levorotatory, which are different from that of the standard heparin from the mammalian source (48°), possibly due to the presence of L-fucose branches. The glucan (HE-3) appears to be dextrorotatory (170.2°), similar to the dextran (182.5°), indicating that the glucan may be a D sugar on the Fischer projection. In addition, the specific rotations of the FuCSs from −37.9° and −56.2° decrease with the drop in molecular weight (Table 1). This was also observed in our previous study on the FuCS from *T. ananas* [10]. 

#### 2.2.3. Molecular Weight and Molecular Weight Distribution

As shown in Table 1, molecular weights of the three types of polysaccharides are different from each other. Molecular weights of the sulfated fucans are around nine-fold of those of the FuCSs, but the FuCSs from different species of sea cucumbers have similar molecular weights [10,18]. The molecular weight of the glucan (HE-3) is also very high (>253 kDa). The molecular weight distribution (*M_w_*/*M_n_*) is used to measure the width of molecular weight distribution, which represents the homogeneity and dispersibility of the polymer. The distribution data indicates that the natural polysaccharides are homogeneous [23].

#### 2.2.4. Intrinsic Viscosity

Viscosity measurements were made on all sodium salt samples in a 0.1 M aqueous NaCl at 25 °C using conventional capillary viscometers of the Ubbelohde type. The intrinsic viscosity ([η]) can be determined by measuring the viscosity of solutions at low concentrations, and then extrapolating to infinite dilution according to the Huggins or Kraemer relationships [24], respectively:

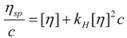
(1)

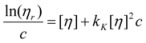
(2)
where η_r_ is the relative viscosity (the ratio of solution viscosity and solvent viscosity η/η_s_), η_sp_ is the specific viscosity (η_r_ − 1), and *k_H_* and *k_K_* are the Huggins coefficient and Kraemer coefficient, respectively.

**Figure 3:**
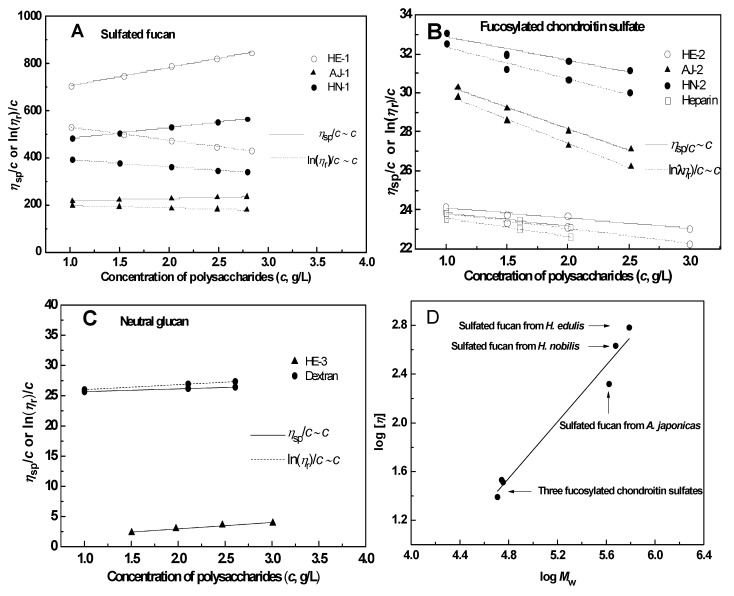
Plots of reduced viscosity (η_sp_/*c*) or logarithmic viscosity (ln(η_r_)/*c*) *vs.* concentrations (*c*) of sulfated fucan (*Panel **A***), fucosylated chondroitin sulfate (*Panel **B***) and neutral glucan (*Panel**** C***). *Panel D* shows relationships between the intrinsic viscosity ([η]) and weight average molecular weight (*M_w_*) of the sulfated polysaccharides.

According to Equations 1 and 2, the intrinsic viscosity [η] was determined by Huggins plots and Kraemer plots with good linear regressions (*R*^2^ > 0.96) (Figure 3A,B,C). The intrinsic viscosities [η] of the three types of polysaccharides were found to be obviously different (Table 1). The intrinsic viscosities of the sulfate fucans are about eight-fold higher than those of the FuCSs, whereas the value of the glucan (HE-3) is fairly low in the same solution (0.8 mL/g), although it also has a very high molecular weight (>253 kDa). Furthermore, Huggins and Kraemer constants (*Κ_H_* and *Κ_K_*) also reflect the various viscosity properties.

In contrast to the sulfated fucans, the three FuCSs from different species of sea cucumbers have similar intrinsic viscosities, possibly due to their similar molecular size and chemical structures [9]. Additionally, comparison analysis of the three types of polysaccharides indicates that the absence of negative charge could result in the low intrinsic viscosity of the neutral glucan. 

Intrinsic viscosity [η] can be related to the weight average *M_w_* of the polymer through the Mark-Houwink-Sakurada (MHS) relationship [10]. A good linear relationship (Figure 3D) was observed between log *M_w_* and log [η] for six different *M_w_* of the sulfated polysaccharide solutions, but not the neutral glucan, and the mathematical equation, log [η] = 1.16 log *M_w_*− 4.02, was obtained by a linear regression (*R*^2^ = 0.973). Thus, the MHS parameters, *K* and α are given as *K* = 9.51 × 10^−5^ mL/g and α = 1.16, respectively. The MHS constants are close to those for the chondroitin sulfate and dermatan sulfate in 0.2 M aqueous NaCl (*K* = 5 × 10^−4^ mL/g, α = 1.14) [25] and those for the FuCS from the sea cucumber *T. ananas* in 0.1 M aqueous NaCl (*K* = 2.406×10^−5^ mL/g, α = 1.305) [10]. The comparatively high value of α in the present study indicates that the structures of the sea cucumber sulfated polysaccharide chains may be intermediate between that of an open coil and a stiff rod; in theory the exponent for a coil should be 0.8 and for a rod 1.8 [25].

### 2.3. NMR Analysis

**Figure 4:**
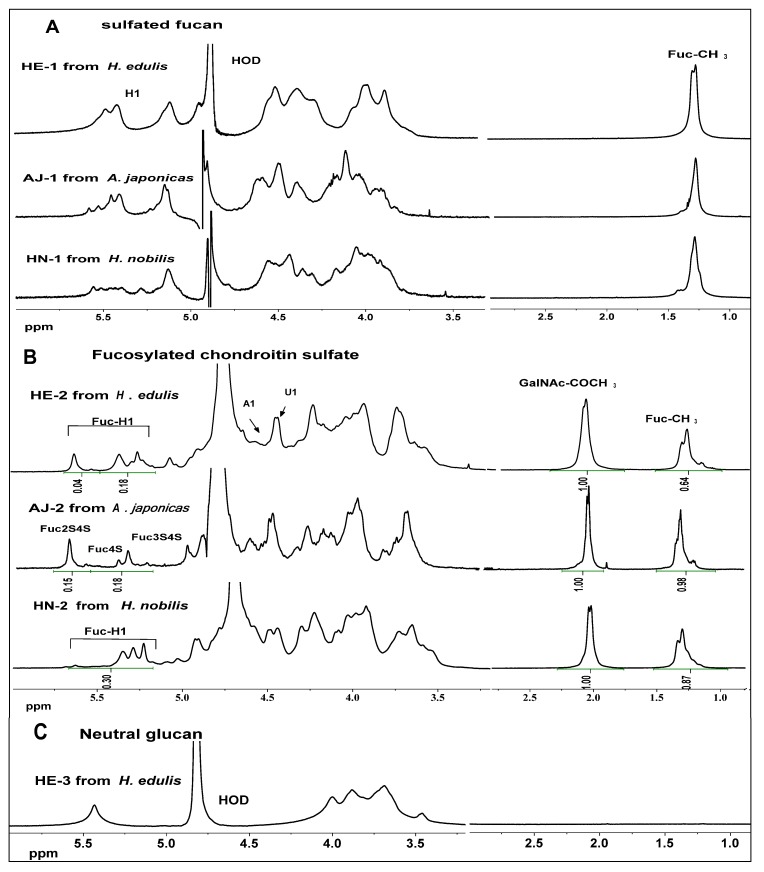
^1^H NMR spectra of the polysaccharides, three sulfated fucans (*Panel **A***), three fucosylated chondroitin sulfates (*Panel **B***) and a neutral glucan (*Panel **C***) from three sea cucumbers.

In order to compare differences between the fine structures of the sulfated fucans, FuCSs and neutral glucan, their ^1^H NMR spectra are shown in Figure 4A,B,C, respectively. In Figure 4A, the signals at about 1.20–1.30 ppm can be readily assigned to the methyl protons of fucose residues (CH_3_). In addition, the chemical shifts of the envelope of anomeric signals at 5.0–5.7 ppm (Figure 4A) are consistent with the presence of α-L-fucopyranosyl units [15,16]. However, the spectra of different species fucans are quite different from each other. Anomeric signals in the spectra of the three sea cucumber fucans are spread from 5.7 to 5.2 ppm, including signals at 5.6 and 5.7 ppm that have no counterpart in any of the other fucans in this study. A feature at around 5.10 ppm can tentatively be assigned to H1 of nonsulfated fucose residues [15]. The sulfated fucans give overlapping spectra with broad signals hampering resolution, since line widths are several Hz, as expected for polysaccharides of high molecular mass. Attempts to record two-dimensional NMR spectra for these polysaccharides gave no useful result in this study (not shown).

Although the FuCSs also give complex spectra, their several individual signals can easily be resolved, based on comparison with relatively much literature data for different species of sea cucumbers [10,13,17,18,19,20,21]. The signals at about 1.20–1.30 ppm and 2.0–2.10 ppm can be readily assigned to the methyl protons of fucose (CH_3_) and *N*-acetyl-β-D-galactosamin (CH_3_CO) residues, respectively (Figure 4B). The shifts from 5.1 to 5.6 ppm may be the anomeric protons of distinctive sulfated fucose residues [13,17,18,19,20,21] and an obvious difference can be observed in this region. The three major signals at 5.69, 5.41 and 5.35 ppm in the spectra of the *A. japonicas* FuCS, similar to those in the *H*. *edulis* FuCS, can be assigned to 2,4-*O*-disulfated fucose (Fuc2S4S), 4-*O*-monosulfated fucose (Fuc4S) and 3,4-*O*-disulfated fucose (Fuc3S4S), respectively (HE-2 and AJ-2 in Figure 4B). The results are in agreement with the signals found in the spectra of the fucosylated chondroitin sulfate isolated from the sea cucumber *S. japonicas* [18]. According to the integrals of the anomeric protons, the contents of Fuc2S4S in HE-2 and AJ-2 are about 18% and 45%, respectively (Figure 4B). But the anomeric proton signals of the *H*. *nobilis* FuCS are rather weak and complicated (HN-2 in Figure 4B). Anomeric signals in the spectra of the *H*. *nobilis* FuCS are spread from 5.49 to 5.29 ppm, with significant differences from all of the other FuCSs in this study. Obviously, the *H*. *nobilis* FuCS does not show the signals at 5.69 ppm (HN-2 in Figure 4B), indicating the absence of Fuc2S4S. The peaks at 5.29 ppm are from Fuc3S, and those at 5.33 and 5.43 ppm are from Fuc3S4S and Fuc4S, respectively, based on the published reports of FuCSs obtained from other species of sea cucumbers such as *T. ananas*, *S. japonicas* and *L. grisea* [13,17,18]. Additionally, different sulfation patterns of the fucose branches also affect the chemical shifts of methyl protons of fucose (CH_3_) and *N*-acetyl-β-D-galactosamin (CH_3_CO), as has been noted [19]. 

**Figure S2:**
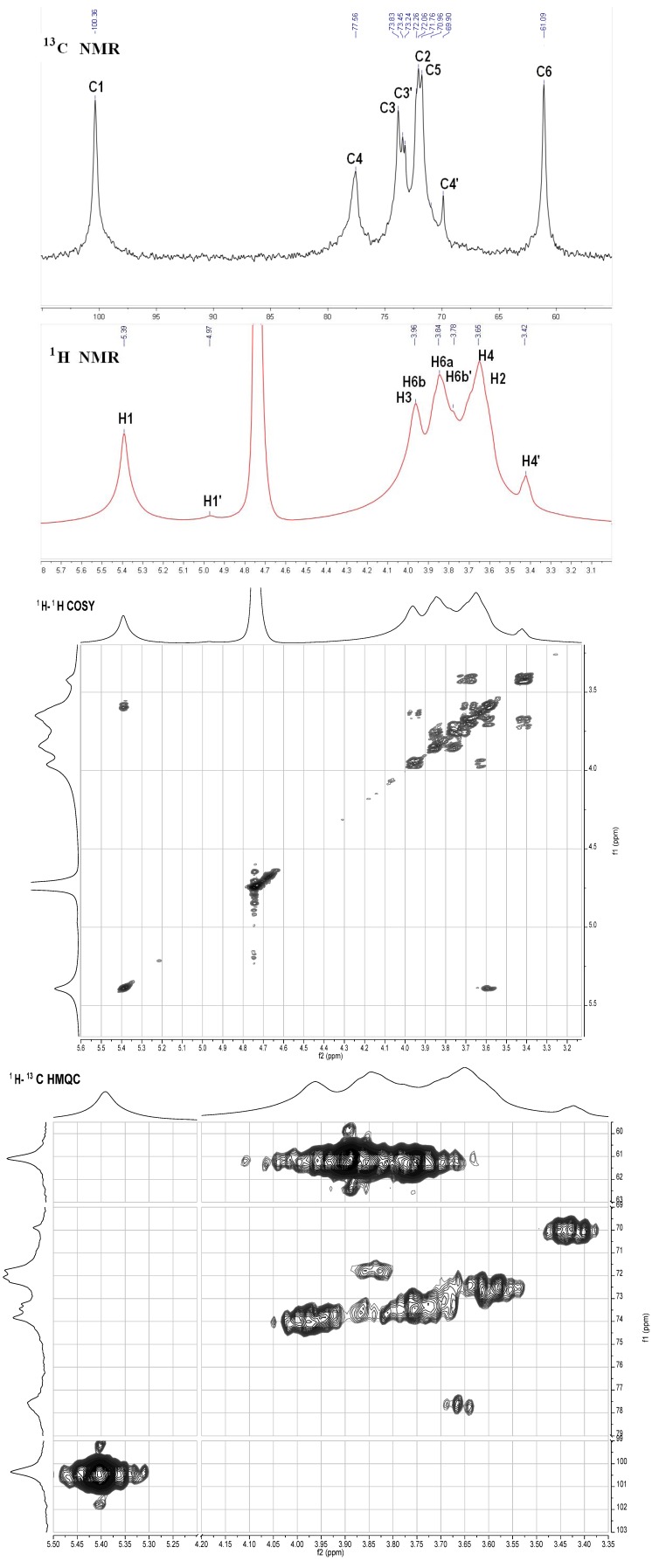
Parts of the spectra of the neutral glucan (^13^C, ^1^H, ^1^H–^1^H COSY, ^1^H–^13^C HMQC).

In contrast to those of the sulfated polysaccharides, the sea cucumber neutral glucan (Figure 4C) gives ^1^H spectrum, which is simple, and although line widths are several Hz, as expected for polysaccharides of high molecular mass, its signals can be assigned. The anomeric proton shift is 5.39 ppm, possible indicating that the glucan may be an alpha sugar. These ^13^C signals 100.36, 77.56 (69.90), 73.83 (73.45), 72.06, 70.96 and 61.09 ppm of the neutral glucan can be readily assigned to the C1, C4, C3, C2, C5 and C6, respectively, based on the interpretations of ^1^H–^1^H correlation spectroscopy, (COSY) and ^1^H–^13^C heteronuclear Multiple-Quantum Correlation (HMQC) spectra (Figure S2) and reported data [26]. In addition, based on the HMQC spectrum, comparison of the chemical shifts for each carbon and proton with shifts for standard glucose showed strong downfield shifts of some signals, indicating the existence of glycosidic (1→4)- and (1→6)- linkages [26]. Thus, NMR analysis preliminarily indicates that this neutral glycan is likely to be an α-(1→4)-D-glucan with a (1→6)-linkage branch (Figure S2), which is consistent with the monosaccharide composition analysis of the fraction HE-3 (Table 1). The neutral glucan was firstly obtained from sea cucumbers, although it has been isolated from mussels [26]. 

### 2.4. Anticoagulant Activity

**Figure 5:**
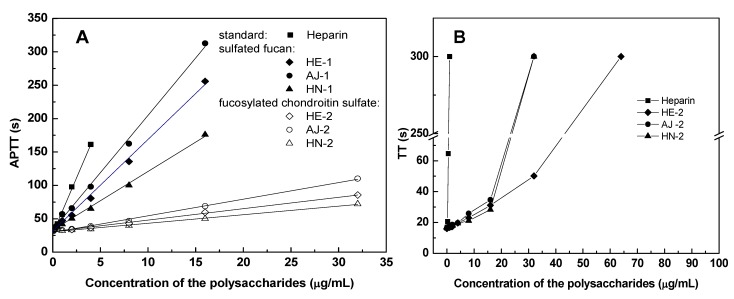
Anticoagulant activities, activated partial thromboplastin time (APTT) (*Panel **A***) and thrombin time (TT) (*Panel **B***), of the polysaccharides from different species of sea cucumbers.

Anticoagulant activities of the polysaccharides from the three sea cucumbers were assessed by measuring the activated partial thromboplastin time (APTT), prothrombin time (PT) and thrombin time (TT), and compared with the same activities of unfractionated heparin from mammalian source. The APTT and TT assays (summarized in Table 2, Figure 5) indicate that the three FuCSs have significant anticoagulant activities, lower than heparin, but much higher than the sulfated fucans (TT of the sulfated fucans is not shown in Figure 5B), although the molecular sizes of the FuCSs are much lower than those of the sulfated fucans (Table 1). The result is highly consistent with that from previous studies on the other FuCS and sulfated fucan from the sea cucumber *L. grisea* (40 U/mg for FuCS *vs.* 2 U/mg for sulfated fucan) [17,27]. But the neutral glucan has no anticoagulant activity, as expected from the absence of sulfate [12]. Thus, our studies have suggested that anticoagulant activities of the different types of polysaccharides are likely to relate to monosaccharide composition and sulfate content. Additionally, the PT assays were also carried out but none of these polysaccharides showed any effects on PT at the concentrations tested (1–1000 μg/mL). The absence of PT activities of the sulfated polysaccharides is similar to that of other sulfated fucans and FuCSs [14,19,28].

**Table 2:** Anticoagulant properties of the polysaccharides purified from different sea cucumbers.

Samples	Sources	APTT ^a^		TT ^a^		PT ^a^	
		μg/mL	U/mg	μg/mL	U/mg	μg/mL	U/mg
**Sulfated fucans**							
HE-1	*Holothuria* * edulis*	18.71	13	>128	<1	>1000	<1
AJ-1	*Apostichopus* * japonicas*	13.30	19	>128	<1	>1000	<1
HN-1	*Holothuria* * nobilis*	26.59	9	>128	<1	>1000	<1
**Fucosylated** ** chondroitin sulfates**							
HE-2	*Holothuria* * edulis*	2.86	89	16.03	6	>1000	<1
AJ-2	*Apostichopus* * japonicas*	2.20	116	14.20	7	>1000	<1
HN-2	*Holothuria* * nobilis*	4.33	59	22.66	4	>1000	<1
**Neutral glucan**							
HE-3	*Holothuria* * edulis*	>1000	<1	>1000	<1	>1000	<1
Standard glycosaminoglycan							
Heparin	Porcine intestine	1.20	212	0.45	212	24	212

^a^ The activity of the polysaccharides to prolong APTT, TT or PT is expressed by the concentration of each agent that is required to double the APTT, TT or PT (doubling APTT, TT or PT, μg/mL), and also is expressed as USP units/mg (U/mg) using a parallel standard curve based on the Heparin 212 units/mg from Sigma (USA).

Interestingly, as shown in Table 2, the anticoagulant activities of the fucosylated chondroitin sulfates from different species of sea cucumbers are found to be obviously different. The *A. japonica* FuCS, with higher Fuc2S4S residues (45% for AJ-2 *vs.* 18% for HE-2, Figure 4B), has higher activity than the other FuCSs (116 U/mg for AJ-2 *vs.* 89 U/mg for HE-2, Table 2). Their sulfation patterns and contents may result in the difference in anticoagulant activities of the FuCSs, since they have similar chemical structures and molecular sizes. In addition, comparison of the NMR data of the FuCSs from three species of sea cucumbers, together with our previous study [13] and a literature report [19], indicates that this potent effect may be related to the occurrence of 2,4-*O*-disulfated fucose units.

## 3. Experimental Section

### 3.1. Extraction and Purification of Polysaccharides

The polysaccharides were extracted from the body wall of sea cucumbers as the previous studies [10,16,17]. The tissue of the dried body wall was digested by 0.5 M sodium hydroxide, and then core protein combined with polysaccharides was released by the papain (EC 3.4.22.2). The crude polysaccharides were purified by gel filtration with a Sephadex G-100 (2 cm × 100 cm, GE Healthcare Technology, Uppsala, Sweden) and ion-exchange chromatography with a DEAE-sephadex FF column (3 cm × 7 cm, Amersham Biosciences, Uppsala, Sweden). The purity of preparation was assayed by high-performance gel permeation chromatography (HPGPC) using a Agilent technologies1200 series (Agilent Co., USA) apparatus with RID (G1362A) and DAD (G1315D) detectors, equipped with a Shodex OH-pak SB-804 HQ column (8 mm × 300 mm). Chromatographic conditions and procedures were performed according to the previous method [23].

### 3.2. NMR Analysis

^1^H spectra were recorded with a Bruker Advance III 600 spectrometer of 600 MHz equipped with Bruker’s CryoProbes. The NMR experiments were recorded at 21 °C with a spectral width of 12335.5 Hz, an acquisition time of 2.6564 s, a pulse width of 7.8 s, a relaxation time of 1 s and a number of 8 scans. All lyophilized polysaccharides were dissolved in deuterium oxide (D_2_O, 99.9% D) at a 3–5 mg/mL concentration. Expansion of the 5.8 to 5.1 ppm region of the spectra of the fucosylated chondroitin sulfates can be readily assigned to the anomeric protons of distinctive sulfated fucose residues [17,18,19,20,21]. The integrals are normalized to a total of 100 = protons. Thus, the content of a sulfated fucose residue can be calculated as a percentage of the total sulfated fucose residues.

### 3.3. Chemical Composition Analysis

Hexuronic acid was measured as described previously [29]. The ratio of GalNAc and Fuc was obtained by ^1^H NMR analysis integrals of their methyl protons (also see Figure 4B). Acetylaminohexose was carried out as described previously [17]. After strong acid hydrolysis of the polysaccharide (4.0 M HCl, 100 °C for 6 h), total hexosamine was estimated by a modified Elson-Morgan reaction [30]. The sulfate/carboxyl groups of the polysaccharides were determined by a conductimetric method [22]. 

### 3.4. Analysis of Molecular Weight

The molecular mass, weight-average molecular mass (*M_w_*), number-average molecular mass (*M_n_*) and molecular weight distribution (*M_w_*/*M_n_*) of polysaccharides were estimated by HPGPC. Chromatographic conditions and procedures were performed according to the previous method [23]. For molecular weight estimation, the columns were calibrated by standard D-series Dextrans (D-0, 2, 3, 4, 5, 6, 7, 8 and 2000), a FuCS from the sea cucumber *T. ananas* with known relative molecular weight (*M_w_* 65,820 Da) for the FuCS and sulfated fucan, a dextran from *Leuconostoc* spp. with relative molecular weight (*M_w_* 100,000 Da) for the neutral glucan.

### 3.5. Specific Rotation

The specific rotation was determined by the optical rotation in *Pharmacopoeia of the People’s Republic of China* [31]. In the method, the concentration of the polysaccharides was about 10 mg/mL and the detection temperature was 20 °C.

### 3.6. Measurement of Intrinsic Viscosity

The Intrinsic viscosity was measured by the method of determination of viscosity in *Pharmacopoeia of the People ’s Republic of China* [32]. 

### 3.7. Anticoagulant Action Measured by APTT, PT and TT

Activated partial thromboplastin time (APTT), prothrombin time (PT) and thrombin time (TT) clotting assays were carried out as described previously [14], using a coagulometer (TECO MC-4000, Germany).

## 4. Conclusions

In summary, three types of polysaccharides from different species of sea cucumbers were isolated and purified, and their physicochemical characteristics, such as chemical composition, electric charge, average molecular weight and intrinsic viscosity, were characterized by different methods. The polysaccharides obtained from sea cucumber are three FuCSs, three sulfated fucans and a neutral glucan, respectively. The three types of polysaccharides have obviously different physicochemical characteristics. However, more attempts to elucidate further refinement of the structure of the sulfated polysaccharides are necessary. 

Comparison analysis of anticoagulant activities of the polysaccharides indicates that anticoagulant activities of the different types of polysaccharides are likely to relate to monosaccharide composition and sulfate content, and the sulfation patterns of the FuCSs may result in a difference in anticoagulant activities. Our data may be of help to elucidate the structure-activity relationship of the sea cucumber polysaccharides.

## Supplementary Material

### S1. Qualitative Analysis of Monosaccharide Composition of the Sea Cucumber Polysaccharide Samples by HPLC

#### S1.1. Materials

The sea cucumber polysaccharide samples were prepared from three sea cucumbers. The monosaccharides including glucuronic acid (GlcA), glucose (Glc), galactose (Gal) were purchased from Alfa Aesar. The *N*-acetylgalactosamine (GalNAc) was purchased from TCI and fucose (Fuc) was from sigma Chemical Co. (Shanghai, China). The 1-phenyl-3-methyl-5-pyrazolone (PMP, 99%) was purchased from ACROS organics.

#### S1.2. Methods

##### S1.2.1. Hydrolysis and PMP Labeling

The dried polysaccharides (5 mg) were dissolved in 1 mL of 2 M trifluoroacetic acid (TFA) in a hydrolysis tube [33]. The hydrolysis tube was sealed and incubated at 100 °C for 8 h in a heating block. After reaction, the reaction mixture was evaporated to dryness. The residue was then dissolved in methanol and again evaporated to dryness to remove residual TFA. This step was repeated four more times. The polysaccharide samples prepared as described above, and the standard samples were reconstituted in deionized water [34]. Then 50 μL of the sample solution and 50 μL of 0.5 M PMP in methanol and 50 μL of 0.6 M sodium hydroxide were added to the mixture. The reaction mixture was mixed and incubated at 70 °C for 30 min in a heating block. After adjusting the pH to 7 by adding 50 μL of 0.3 M HCl, 1 mL of chloroform was added to the mixture and mixed for at least 5 s using a vortex mixer. The bottom chloroform layer was carefully removed using a pipe and discarded. This step was repeated three more times, and the top aqueous layer was saved for HPLC analysis.

##### S1.2.2. HPLC Analysis

Analysis of the PMP-labeled polysaccharides was carried out using an Agilent technologies1200 series (Agilent Co., USA) apparatus with DAD (G1315D) detectors, equipped with an Agilent Eclipse XDB C18 (150 mm × 4.6 mm). The ﬂow rate was 1 mL/min, and UV absorbance of the effluent was monitored at 250 nm. Buffers A and B were 0.1 M ammonium acetate, pH 5.5, containing 22% acetonitrile, respectively.
